# Differential Sensing of Saccharides Based on an Array of Fluorinated Benzosiloxaborole Receptors

**DOI:** 10.3390/s20123540

**Published:** 2020-06-22

**Authors:** Paweł Ćwik, Patrycja Ciosek-Skibińska, Marcin Zabadaj, Sergiusz Luliński, Krzysztof Durka, Wojciech Wróblewski

**Affiliations:** 1 Chair of Medical Biotechnology, Faculty of Chemistry, Warsaw University of Technology, Noakowskiego 3, 00-664 Warsaw, Poland; cwieku@gmail.com (P.Ć.); mzabadaj@ch.pw.edu.pl (M.Z.); wuwu@ch.pw.edu.pl (W.W.); 2 Chair of Physical Chemistry, Faculty of Chemistry, Warsaw University of Technology, Noakowskiego 3, 00-664 Warsaw, Poland; serek@ch.pw.edu.pl (S.L.); kdurka@gmail.com (K.D.)

**Keywords:** molecular receptors, benzosiloxaboroles, chemometric analysis, saccharide discrimination, pattern-based sensing

## Abstract

Fluorinated benzosiloxaboroles–silicon congeners of benzoxaboroles, were synthesized and tested as molecular receptors for mono- and disaccharides. The receptors differed in the Lewis acidity of the boron center as well as in the number of potential binding sites. The calculated stability constants indicated different binding affinity of benzosiloxaborole derivatives towards selected saccharides, enabling their classification using a receptor array-based sensing. Unique fluorescence fingerprints were created on the basis of competitive interactions of the studied receptors with both Alizarin Red S (ARS) and tested saccharide molecules. Detailed chemometric analysis of the obtained fluorescence data (based on partial least squares-discriminant analysis and hierarchical clustering analysis) provided the differential sensing of common saccharides, in particular the differentiation between glucose and fructose. In addition, DFT calculations were carried out to shed light on the binding mechanism under different pH conditions.

## 1. Introduction

The synthesis of a parent benzoxaborole has been reported already in the 1950s [1,2]. However, the strong interest in this group of boraheterocycles emerged only in the previous decade and was stimulated by the discovery of their potent antimicrobial activity [3]. Extensive studies resulted in the preparation of numerous derivatives which were further evaluated from the point of view of medicinal chemistry [4]. Those efforts have already met with success as two benzoxaboroles including antifungal agent tavaborole (trade name Kerydin) and anti-inflammatory crisaborole (trade name Eucrisa) were approved by FDA for the clinical use [5]. The mechanism of action of benzoxaboroles relies on their physicochemical specificity based on enhanced electron-deficient character of the boron atom. In fact, benzoxaboroles are stronger Lewis acids than the corresponding arylboronic acids [6]. This results not only in improved solubility in water at neutral pH but also may be beneficial for ability to bind various cis-1,2-diols, including biologically important saccharides, nucleosides, and catechol derivatives [7,8]. The importance and potential wide applicability of benzoxaboroles has prompted us to develop their analogues, namely pyridoxaboroles [9] and benzosiloxaboroles [10]. Thus, in the former case the benzene ring of benzoxaborole was replaced with a pyridine one, whereas in the latter the five-membered ring comprising silicon atom was constructed. It should be noted that specific electronic properties of silicon result in further enhancement of the Lewis acidity of the boron atom.

Recently, the synthesis and comprehensive characterization of benzosiloxaboroles bearing various functional groups such as halogens (X = F, Cl, Br), CHO, CH_2_OH, B(OH)_2_ was reported [11]. Antimicrobial activity of those derivatives was evaluated, and the structure–properties relationships were discussed. Interestingly, selected benzosiloxaboroles showed promising inhibitory activity against KPC-2 β-lactamase and the mechanism of action was studied theoretically [12]. Apart from the work devoted to the potential use of benzosiloxaboroles as antimicrobials, we have also performed studies on their affinity to selected molecules bearing cis-1,2-diol moiety. Preliminary results showed that fluorinated benzosiloxaboroles are effective receptors of biologically relevant diols including ribose, adenosine, and dopamine [10].

Classical sensing methods are based on lock-and-key principle, that requires highly selective receptors for detecting specific target analytes. However, as a promising alternative for addressing the deficiencies of this traditional chemosensing approach, differential sensing has emerged [13,14]. This novel analytical strategy involves pattern-based sensing, similar to human olfactory and gustatory systems. It assumes application of cross-reactive semi-selective receptors, organized in a form of an array, whose responses are analyzed as a whole in order to decode significant analytical information from multidimensional signals [15]. This concept has gained recently much attention, which resulted in the elaboration of a variety of sensing array platforms dedicated to a wide range of analytes, including peptides, proteins, sugars, flavonoids, volatile organic compounds, toxic industrial gases, organic compounds in water, and many others [14,15,16].

Boronic compounds exhibit selectivity towards various diols, however the majority of these molecular receptors for saccharides have generally not shown sufficient degrees of affinity and selectivity in aqueous media [17]. Therefore, boronic compounds are naturally predestined towards pattern-based, differential sensing of sugars and sugar analogues [18]. As an example, a chemosensor array for saccharides and saccharide derivatives, fully operational in aqueous media at physiological pH, was reported [17]. Boronic acid-based peptidic receptors, derived from a combinatorial library, served as the cross-reactive sensor elements, while the binding of saccharides was assessed colorimetrically using an indicator uptake assay performed in a micromachined chip platform. Boronic acid-modified poly(amidoamine) dendrimers were also applied as sugar-sensing materials, for the discrimination of simple sugars in water (fructose, glucose, galactose, and ribose); a pattern-based sensing array was developed using multiple combinations of the two dendrimeric receptors, two dyes, and two pH conditions [19]. An indicator displacement assay, based on an anionic fluorescent dye (8-hydroxypyrene-1,3,6-trisulfonic acid trisodium salt) and an array of six cationic diboronic acid appended benzyl viologens was developed for the discrimination of 12 saccharides [20]. Similar fluorescent probe array was applied to the discrimination of sugar and sugar alcohols of biomedical relevance [21]. Another example of a pattern-based assay based on boronic compounds is a library of diboronic receptors with various spacers that were synthesized for the sensing of ginsenosides. The incorporation of two boronic acid groups allowed the pairing of two indicators. The resulting dynamic three-component sensing ensembles were capable of responding differently to ginsenosides, revealing excellent identification of these compounds [22].

In this work, fluorinated benzosiloxaborole derivatives of various structure and boron center acidity were proposed as molecular receptors for saccharides. The discrimination of selected mono- and disaccharides was attempted based on different binding properties of the studied benzosiloxaborole derivatives and chemometric analysis.

## 2. Materials and Methods

### 2.1. Structures of Fluorinated Benzosiloxaboroles Receptors **1**–**5**

**Figure 1:**
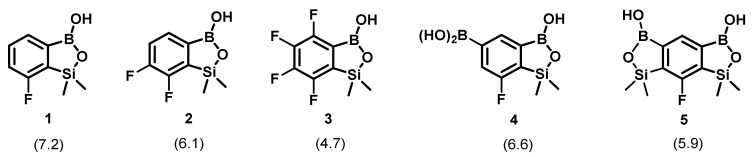
Structures of fluorinated benzosiloxaboroles **1**–**5** (the p*K*a values determined in [12] are given in parentheses).

The synthesis of fluorinated benzosiloxaboroles **1** and **3** (Figure 1) was described in our previous paper on this group of compounds [10] and discussed in detail in a subsequent publication [23]. Compounds **2**–**5** were prepared analogously using procedures reported recently [12]. Compounds **1**–**3** are simple benzosiloxaboroles which differ only in a number of fluorines at the benzene ring whereas **4** and **5** comprise two boron centers. The acidity of **1**–**5** is significantly higher when the extent of fluorination is increased, the presence of an additional boronic group exerts a similar effect (the p*K*_a2_ value was determined for **4** and **5**).

### 2.2. Determination of the Stability Constants of Benzosiloxaborole Complexes

The values of stability constants of studied benzosiloxaborole complexes with selected target compounds (saccharides) were assessed by the method introduced by Springsteen and Wang, utilizing Alizarin Red S (ARS) as a fluorescent probe [24]. A series of solutions with the fixed concentration of ARS (9 μM) and various concentrations of respective benzosiloxaborole receptors **1**–**5** (up to 0.45 mM), i.e., various molar ratios of receptors to ARS, were prepared in 0.05 M HEPES buffer pH 7.0. For every solution four separate wells in a 96-well plate were filled and subjected to spectroscopic measurements independently. The fluorescence spectra were measured in 500–700 nm range (excitation wavelength 450 nm) with 1 nm steps, using a Synergy Mx Microplate Reader (BioTek Instruments Inc., Winooski, VT, USA). The stability constants of benzosiloxaborole-ARS complexes K_1_ were calculated according to the Benesi–Hildebrand method (maximum fluorescence intensities recorded at 590 nm were taken into calculations, every point was an average of measurements made for four individual solutions). The stability constants of benzosiloxaborole-saccharide complexes K_s_ were determined by the titration of respective benzosiloxaborole-ARS complex solution with a selected saccharide. A series of solutions with the fixed concentration of receptors **1**–**5** (0.45 mM), ARS (9 μM), and a range of saccharide concentrations were prepared in 0.05 M HEPES buffer pH 7.0 (the added portions of saccharide solutions were tuned to reduce the fluorescence intensity to the level of 25−35% of the value measured for an original solution). The fluorescence spectra were recorded as described above (each point taken for calculations was an average of four individual measurements). Detailed procedures used for the calculation of K_1_ and K_s_ stability constants were described in [10].

### 2.3. Determination of the Stoichiometry of Formed Complexes

Stoichiometry of receptor-ARS complexes was determined for benzosiloxaboroles **4** and **5**. The classical spectrophotometric method of continuous variations (Job’s method) was applied. A series of 16 solutions with varying molar ratios of ARS to receptor was prepared in 0.05 M HEPES buffer pH 7.0 (the sum of concentration of both components was 1 mM in every solution). The UV-Vis spectra were measured at 250–800 nm with 1 nm steps, using Varian Cary 60 Spectrophotometer (Agilent Technologies). The values of absorbance recorded at 450 nm were plotted against ARS to receptor ratio, providing the estimation of the stoichiometry of formed complexes.

### 2.4. Chemometric Analysis

The data for chemometric analysis were obtained as follows: a series of solutions containing 9 μM ARS, 0.45 mM of respective receptor **1**–**5** as well as 2, 10, or 25 mM of target compound (saccharide) was prepared in 0.05 M HEPES buffer pH 7.0. For every solution eight separate wells in a 96-well plate were filled and subjected to spectroscopic measurements independently. The emission spectra were measured in 500–700 nm range (excitation wavelength 450 nm) with 1 nm steps, using Synergy Mx Microplate Reader (BioTek Instruments Inc.). Data analysis was performed in MatLab (The MathWorks, Inc., Natick, MA, USA) with the PLS Toolbox (EigenvectorResearch Inc., Wenatchee, WA, USA) and Origin (Microcal Software, Inc., Northampton, MA, USA) software.

### 2.5. Computational and NMR Spectroscopy Studies

The single-molecule geometry optimizations were performed with DFT method using B3LYP functional [25,26] and 6-311 + G(d,p) basis-set [27] implemented in Gaussian16 program. The minima were confirmed by vibrational frequency calculations within the harmonic approximation (no imaginary frequencies). During the calculations no symmetry constraints were applied. A conductor-like polarizable continuum model (CPCM) with water was used to imitate solvent effect. NMR spectra were recorded on a 400 MHz Agilent VnmrS NMR spectrometer. ^1^H NMR chemical shifts are given relative to TMS using residual solvent (DMSO-d_6_) resonances. ^19^F NMR chemical shifts are given relative to CFCl_3_.

## 3. Results and Discussion

Previous studies indicated that the acidity of benzosiloxaboroles has a relevant impact on their ability to bind particular saccharides [10]. Thus, in this work simple fluorinated derivatives **1**–**3** of increasing Lewis acidity of boron atom were chosen. The selection was complemented by two additional compounds (receptors **4** and **5**), to evaluate the influence of the presence of two boron centers potentially able to bind saccharides on the receptor properties with respect to simpler benzo-siloxaboroles **1**–**3**.

### 3.1. Binding Affinity of Benzosiloxaboroles **1**–**5** Towards Saccharides

**Figure 2:**
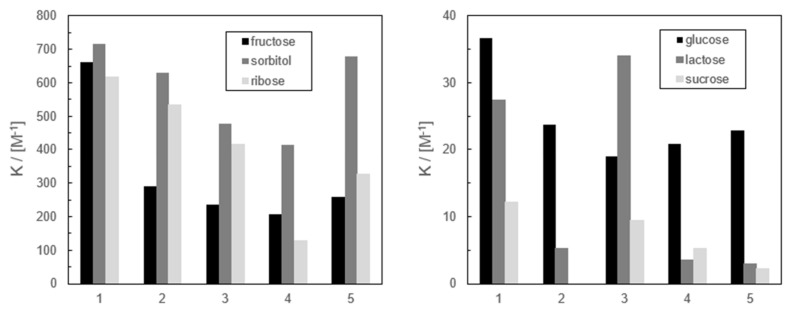
Stability constants of complexes of benzosiloxaboroles **1**–**5** with selected saccharides.

The values of calculated stability constants of complexes of studied benzosiloxaboroles with selected carbohydrates were collected in the form of histograms in Figure 2. In general, certain affinity patterns can be remarked—the most considerable is the ability to bind ARS negatively correlated with the acidity of receptors. This dependence can be explained assuming that the physicochemical properties of benzosiloxaboroles are similar to those of benzoxaboroles. According to Benkovic and co-workers benzoxaboroles bind ARS most firmly in their neutral form, while one of the hydroxyl groups of ARS is deprotonated [28]. When the benzoxaborole group binds the hydroxyl anion, repulsion forces between ARS and receptor hinder the binding. A similar tendency is observed for studied benzosiloxaboroles, taking into account the values of binding constants with particular target compounds.

In general, the studied benzosiloxaborole receptors form the most stable complexes with sorbitol, whereas the affinity to the remaining target compounds varied depending on the structure of receptors. The least acidic receptor **1** exhibits the highest binding affinity towards studied saccharides (except the affinity of the most acidic compound **3** to lactose). Moreover, there is no significant correlation between the acidity of receptors **2**–**5** and their binding properties. Initially, we assumed that receptors **4** and **5** will show improved binding ability due to the presence of two boron centers. However, it is clearly not the case, especially for **4** which can be regarded as 6-B(OH)_2_ substituted derivative of **1**. In fact, compound **4** binds glucose and fructose to a similar extent as **2**, **3**, and **5**, whereas its affinity towards ribose and sorbitol is relatively weaker. Thus, it seems that the affinity of the studied benzosiloxaboroles towards saccharides is dependent not only on the acidity of receptors and the number of boron centers in their molecules, but is also significantly influenced by other factors such as their hydrophilicity, tendency to auto-association, or steric factors.

Finally, it should be emphasized that different affinity patterns of the studied receptors towards selected mono- and disaccharides could be useful for the identification of sugars based on an array of benzosiloxaborole derivatives and chemometric analysis.

### 3.2. Stoichiometry of the Complexes Formed by Benzosiloxaborole **4** and **5**

The stoichiometry of receptor-ARS complexes was studied for benzosiloxaboroles **4** and **5** since both derivatives include two binding sites in their structure, enabling binding of two ARS molecules. Classical spectrophotometric Job’s method (method of continuous variations) was applied for this purpose. The Job’s plots revealed that the reaction stoichiometry for the formation of receptor **4**-ARS complex is 1:1, i.e., probably only benzosiloxaborole group contributes to ARS binding. This statement is justified by the fact that the values of stability constants of benzosiloxaborole-ARS complexes are several times higher than the analogue complexes formed by boronic acids. On the other hand, the interpretation of the results obtained in the case of receptor **5** was not so obvious; however, we assumed that ARS molecules bind to both benzosiloxaborole groups.

### 3.3. Theoretical and NMR Spectroscopy Insight into the Mechanism of Complexation of Benzosiloxaboroles with Diols

**Figure 3:**
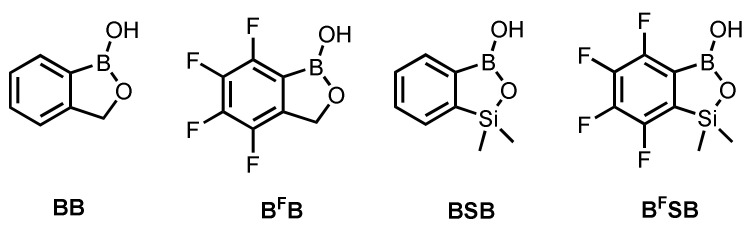
Structures of benzoxa- and benzosiloxaboroles used in computational studies.

DFT calculations on B3LYP/6-311++G(d,p) level of theory were performed in order to shed light on the mechanism of the binding of benzosiloxaboroles to 1,2-diols under alkaline and neutral/acidic conditions. To speed up the calculations, ethylene glycol (EG) was chosen as the simplest 1,2-diol. Furthermore, the effect of acidity was assessed by comparison of the parent benzosiloxaborole **BSB** with its analogue **B^F^SB**, featuring perfluorinated aromatic ring (i.e., receptor **3**). The calculations were also carried out for benzoxaborole **BB** and its 4–7-tetrafluoro analogue **B^F^B** (see Figure 3).

**Figure 4:**
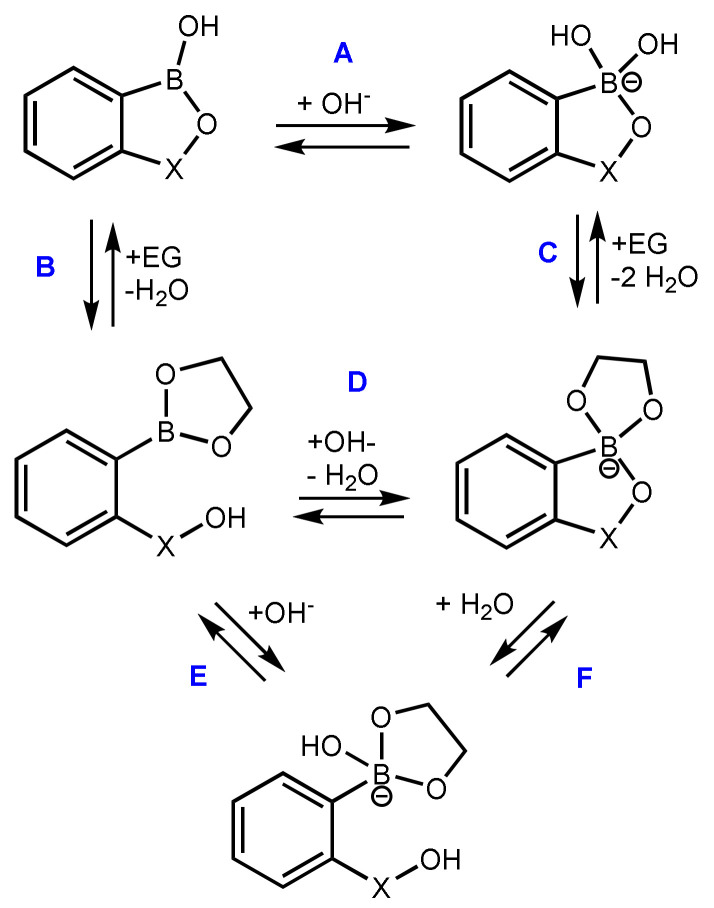
The equilibria for ethylene glycol (EG) binding with benzoxa- (X = CH_2_) and benzosiloxaboroles (X = SiMe_2_).

A set of Δ*G* values (Table 1) characterizing the various equilibria involving neutral and anionic forms of studied compounds as well as their complexes with EG were obtained (Figure 4). The results indicate that boracyclic rings in all analyzed compounds are stable; i.e., they are not prone to hydrolytic cleavage irrespective of the pH. Similarly, binding of EG under acidic conditions resulting in the formation of respective cyclic esters (with concomitant liberation of SiMe_2_OH or CH_2_OH group) is thermodynamically unfavorable (equilibrium B). However, it should be noted that the Δ*G* values for such reactions involving **BB** and **BSB** are only slightly positive (ca. 14 kJ mol^−1^), which means that the complexation of EG might occur to some extent. Since under acidic conditions the formation of anionic complex is not possible, EG can be bonded to boronic group exclusively via oxaborole ring opening. Furthermore, the calculations show that the protonation of oxaborole oxygen atom results in the destabilization of receptor-EG complex, which is manifested by oxaborole B-O bond cleavage. Under alkaline conditions, the complexation of EG proceeds effectively due to negative Δ*G* values (ca. −30 kJ mol^−1^) with the formation of respective *B*-spiro anions (equilibrium C). It should be noted that they are not susceptible to hydrolytic cleavage of siloxaborole/carboxaborole ring (equilibrium F), although it was previously hypothesized that such as a process might be responsible for the decreased ability of benzoxaborole to bind sugars under alkaline conditions [8]. The computational results are in line with ^1^H and ^19^F NMR spectral data of the system **1**+EG in D_2_O/DMSO (3:2 *v/v*) which confirmed that EG is not bound by compound **1** in the absence of a base since the spectra of 1 are not changed at all after addition of the diol (see Supporting Information, Figures S1, S2, S5, and S6). In a slightly alkaline solution (buffered with NaHCO_3_) the anionic form of **1** is formed as indicated by upfield shift of ^1^H NMR signals of aromatic as well as SiMe_2_ protons (Figure S3); similarly, the ^19^F NMR resonance of the fluorine atom is also shifted from −101.09 to −102.80 ppm (Figure S4). The addition of EG to the buffered solution of **1** results in strong broadening of all resonances in the ^1^H NMR spectrum (Figure S7) which clearly indicates that the dynamic binding of EG occurs. In the respective ^19^F NMR spectrum (Figure S8) two broad resonances appear at −102.35 and −103.56 ppm. They may be assigned to two equilibrating forms of a complex **1**-EG and thus we suppose that the *B*-spiro anion exists in the equilibrium with the open structure where the EG is bound to the boron atom in a monodentate fashion. Since this work is not focused on the mechanistic aspects of the diol binding, we did not conduct further in-depth studies, e.g., aimed at estimation of thermodynamic parameters of the process. It should be noted here that NMR spectroscopy is a useful tool for studying the mode of binding and the binding affinities of boronic acid derivatives (including cyclic derivatives such as benzoxaboroles) [29,30,31,32].

The obtained results can be used to elucidate the generally lower binding abilities of receptors **2**–**5** vs. **1** towards studied sugars. At pH = 7.0 more acidic compounds **2**–**5** exist mostly in their anionic forms, whereas for **1** (p*K*_a_ 7.2) there is a higher proportion of the neutral form. Complexation with a diol results in the full conversion of both forms to the anionic *B*-spiro structure due to higher acidity of the complex, compared to free receptor **1**. However, one should bear in mind that the formation of this anion from the neutral receptor molecule is much more advantageous than from its anionic form, which in turn is responsible for higher apparent binding constant values determined for **1**.

### 3.4. Chemometric Analysis

Benzosiloxaboroles bind strongly diol-containing compounds with high affinities via boronate ester formation. In our case, competitive interactions of the studied receptors with both ARS and saccharide molecules were accompanied by different changes in fluorescence spectra. Thus, specific and unique binding-based fluorescence fingerprint can be applied for the classification of the investigated saccharides.

**Figure 5:**
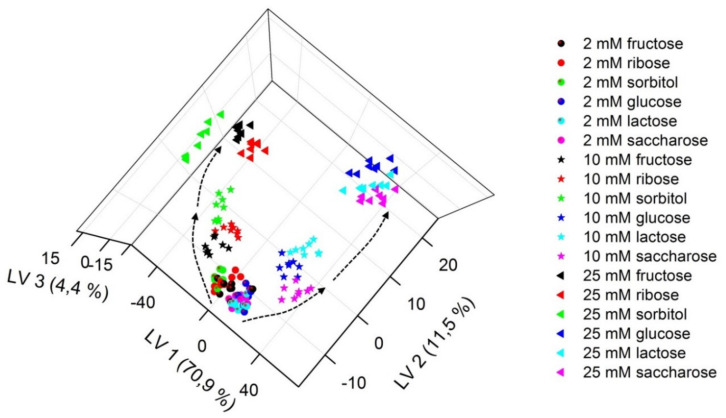
Partial least squares-discriminant analysis (PLS-DA) plot of chemical images of samples containing studied receptors, Alizarin Red S (ARS), and selected saccharides (at three various concentration levels).

Partial least squares-discriminant analysis (PLS-DA) was applied to process the fluorescence data (whole emission spectra) in order to reduce the dimensionality of the data set, to find underlying variables, and to expose the fingerprints more clearly. This numerical procedure is a supervised, multivariate analysis technique aimed at minimizing the separation within the replicates in a group (samples with the same kind of saccharide), while maximizing the distances between groups (samples with various saccharides) using the information from the target matrix on class assignation. One of the products of such analysis is a PLS-DA score plot, which allows for visualization of discrimination ability of the obtained chemical images of the samples. Figure 5 shows the obtained three-dimensional PLS-DA plot of fluorescence data for solutions containing ARS and the studied receptors after the addition of fructose, ribose, sorbitol, glucose, lactose, and sucrose (at three various concentration levels). The fluorescence fingerprints corresponding to samples comprising each saccharide can be clustered into tight distinct groupings (especially when higher concentrations are considered), demonstrating the reproducibility of the analysis. Worse discrimination is observed for samples of low saccharides concentration, due to the low intensity of fluorescence (close to the noise level). It is noticeable that higher order grouping of chemical images can be discernible—the clusters can be collected into two groups in the PLS-DA plot: one composed of glucose, lactose, sucrose; the second one formed by sorbitol, fructose, and ribose. The chemical images of glucose, lactose, and sucrose are close to each other due to their similar chemical structure (glucose is a monosaccharide—an aldohexose, lactose is a disaccharide derived from glucose and galactose, whereas sucrose is also a disaccharide but composed of glucose and fructose). On the contrary, fructose and ribose can form a five-membered furanose ring in solution (in equilibrium with the pyranose structure, typical for glucose), whereas sorbitol does not form a cyclic structure. Thus, the discrimination between two groups of sugars can be observed along the first Latent Variable (LV1), while the second Latent Variable (LV2) allows tracing the changes of saccharide concentrations.

**Figure 6:**
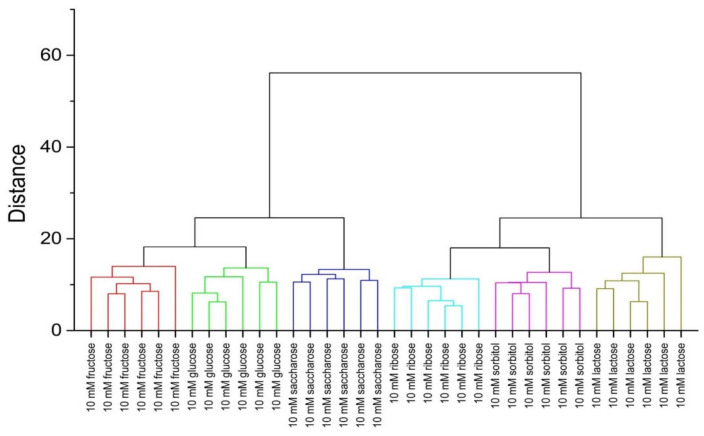
Hierarchical clustering analysis (HCA) dendrogram for samples containing studied receptors, ARS, and saccharides (10 mM).

**Figure 7:**
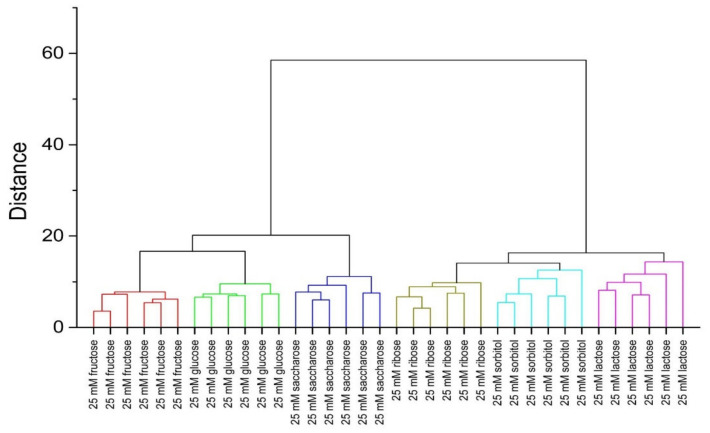
HCA dendrogram for samples containing studied receptors, ARS, and saccharides (25 mM).

The classification ability was investigated in detail to see if the obtained clusters are linearly separable and not overlapping. Hierarchical clustering analysis (HCA) was applied for this purpose. Data provided by PLS-DA treatment were used for the calculations based on Euclidean distance considering the all Latent Variables in the model. HCA was performed separately for two levels of saccharides concentration: 10 and 25 mM; the results are presented in Figure 6 and Figure 7, respectively. Clear and appropriate classification of the solutions containing the investigated saccharides is observed on both dendrograms—the replicates of samples with the same sugar are closest to each other and they form a group placed further from groups assigned to other saccharides. This is evidence of discrimination between each saccharide group and confirms the applicability of fluorescence fingerprints for saccharide recognition and quantification.

## 4. Conclusions

In recent years great progress in the design, optimization, and application of supramolecular pattern-based sensing can be observed [33]. Various semi-selective, cross-responsive receptors for the differential sensing of a variety of analytes were developed, including boronic acid derivatives dedicated especially to the detection, identification, and quantification of sugars and sugar analogues. It was proved, that by mimicking the mammalian olfactory and gustatory systems, differential sensing platforms can provide advancements in sensitivity and pattern-based selectivity above and beyond that what nature provides [18].

In this work, a group of novel fluorinated benzosiloxaborole receptors displaying high affinity towards diol-containing species was characterized for their potential application in selective saccharide recognition. The receptors differed in the Lewis acidity (by the stepwise fluorination of benzene ring) as well as in the number of potential binding sites (by the introduction of an additional siloxaborole ring or boronic acid group). Various affinities of the tested benzosiloxaborole derivatives towards selected saccharides provided the possibility of their identification using differential sensing. High-dimensionality data obtained from the developed receptor array were analyzed using multivariate techniques: PLS-DA and HCA, providing excellent clustering results. The detailed chemometric analysis of the fluorescence data indicated that a group of six common carbohydrates can be discriminated at concentration as low as 10 mM. The proposed differential sensing routine enabled in particular the differentiation between glucose (as well as glucose-based disaccharides) and fructose. In future, boronic acid arrays could be a basis of the development of inexpensive, robust, and rapid analytical tools that play a significant role in high-throughput analysis of sugars or sugar analogues in complex media for biomedical analysis [21]. For such applications, however, first additional studies considering high complexity of biological matrices need to be addressed.

## Figures and Tables

**Table 1:** Gibbs free energy values for processes depicted in Figure 7.

Δ*G*/kJ mol^−1^	BB	B^F^B	BSB	B^F^SB
A	−55.8	−86.3	−59.4	−89.6
B	14.0	20.1	14.2	29.3
C	−30.5	−32.1	−27.9	−27.9
D	−100.3	−138.5	−97.5	−142.8
E	−60.6	−77.1	−62.2	−74.7
F	39.7	61.4	35.3	68.1

## Supplementary Materials

The following are available online at https://www.mdpi.com/1424-8220/20/12/3540/s1, Figure S1: ^1^H NMR spectrum (400 MHz, D_2_O + DMSO-d_6_ 3:2) of **1**., Figure S2. ^19^F NMR spectrum (376 MHz, D_2_O + DMSO-d_6_ 3:2) of compound **1**., Figure S3. ^1^H NMR spectrum (400 MHz, D_2_O + DMSO-d_6_ 3:2) of compound **1** in the presence of NaHCO_3_., Figure S4. ^19^F NMR spectrum (376 MHz, D_2_O + DMSO-d_6_ 3:2) of compound **1** in the presence of NaHCO_3_., Figure S5. ^1^H NMR spectrum (400 MHz, D_2_O + DMSO-d_6_ 3:2) of **1** in the presence of ethylene glycol., Figure S6. ^19^F NMR spectrum (376 MHz, D_2_O + DMSO-d_6_ 3:2) of **1** in the presence of ethylene glycol., Figure S7. ^1^H NMR spectrum (400 MHz, D_2_O + DMSO-d_6_ 3:2) of **1** in the presence of ethylene glycol and NaHCO_3_., Figure S8. ^19^F NMR spectrum (376 MHz, D_2_O + DMSO-d_6_ 3:2) of **1** in the presence of ethylene glycol and NaHCO_3_., Figure S9. Overlay of ^19^F NMR spectra (376 MHz, D_2_O + DMSO-d_6_ 3:2) of **1** in different conditions.
