# Supplementary material for: Differential Sensing of Saccharides Based on an Array of Fluorinated Benzosiloxaborole Receptors
Source: Sensors (Basel). 2020 Jun 22;20(12):3540. doi: 10.3390/s20123540 (PMC7349318; doi:10.3390/s20123540)
Supplement: Supplementary file 1 [file sensors-20-03540-s001.pdf]

Differential sensing of saccharides based on an array of fluorinated benzosiloxaborole receptors

Paweł Ćwik [1], Patrycja Ciosek-Skibińska [1,*], Marcin Zabadaj [1], Sergiusz Luliński [2], Krzysztof Durka [2] and Wojciech Wróblewski [1]

^{1}H and ^{19}F NMR studies on the system **1+**ethylene glycol.

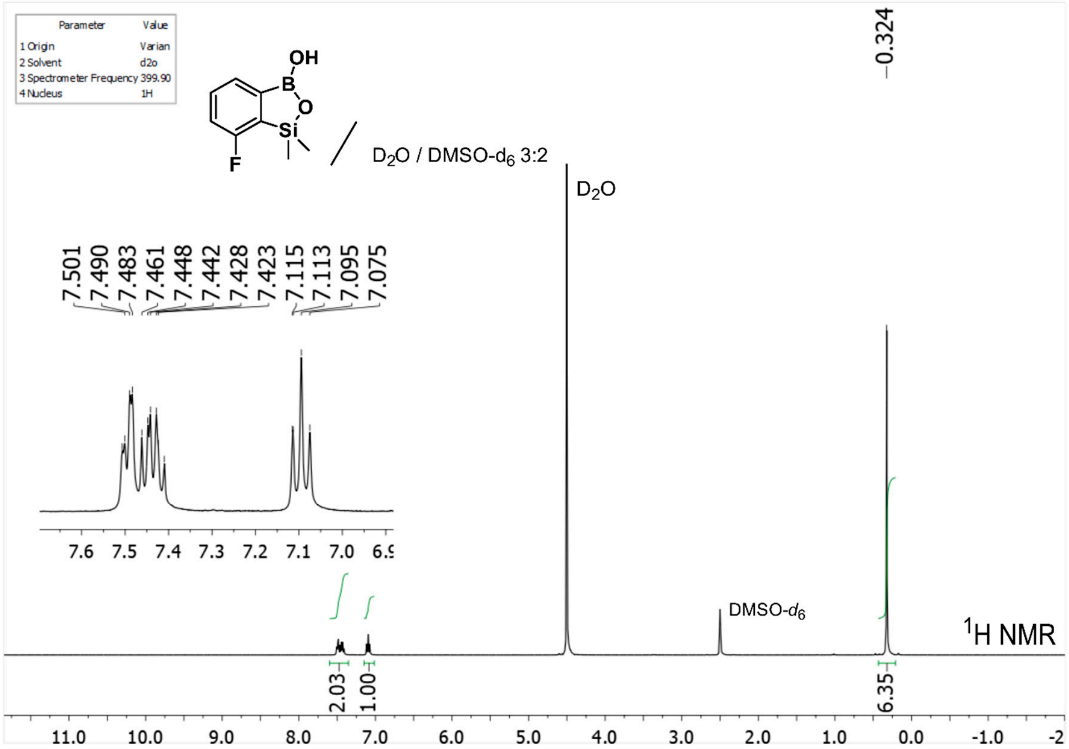

Figure S1. ^{1}H NMR spectrum (400 MHz, D_2O + DMSO-d_6 3:2) of **1**.

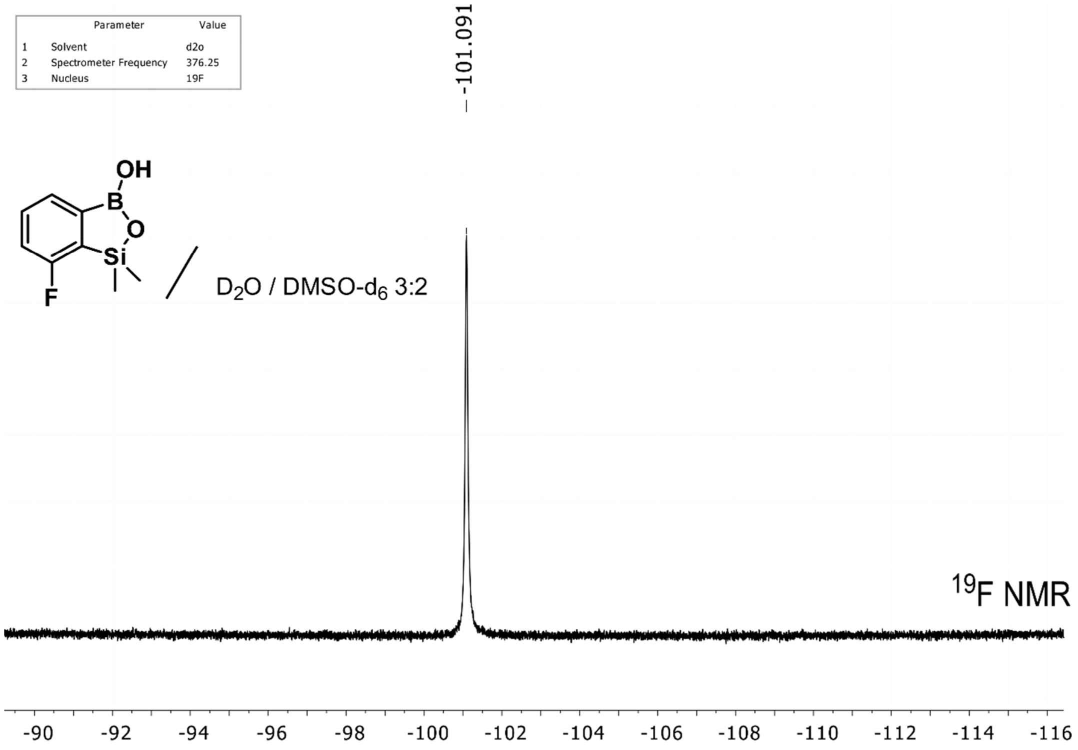

Figure S2. ¹⁹F NMR spectrum (376 MHz, D_2O + DMSO-d_6 3:2) of compound **1**.

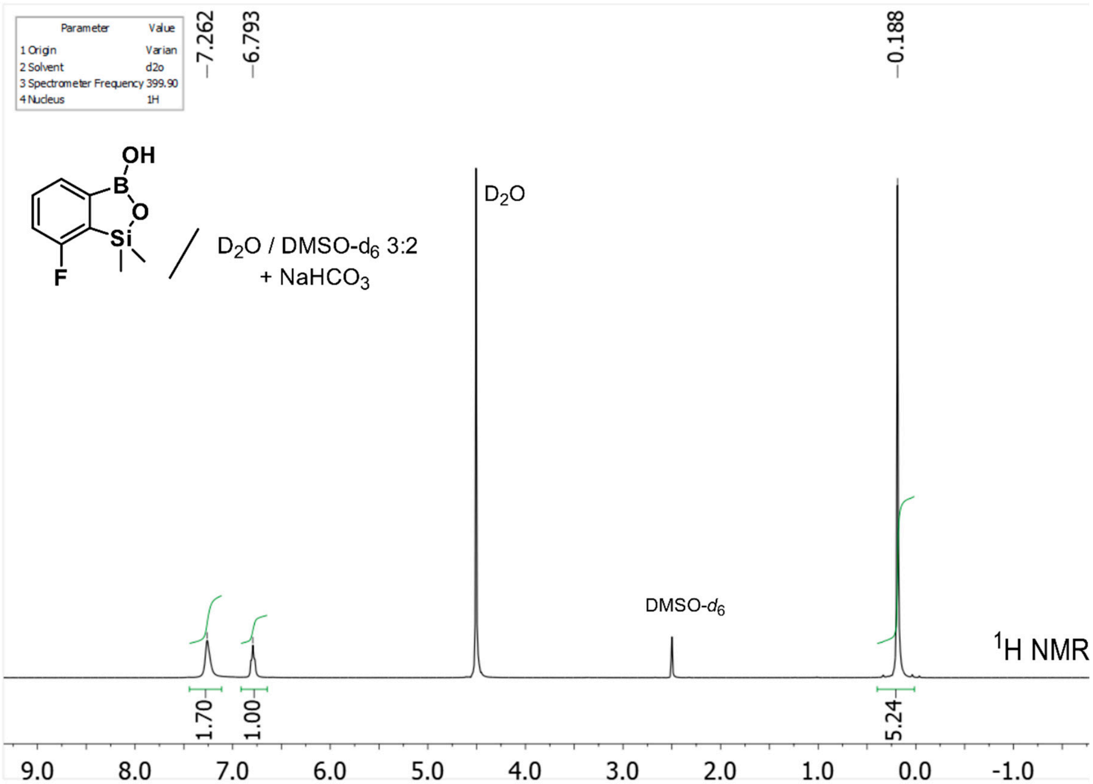

Figure S3. ¹H NMR spectrum (400 MHz, D_2O + DMSO-d_6 3:2) of compound **1** in the presence of $NaHCO_3$.

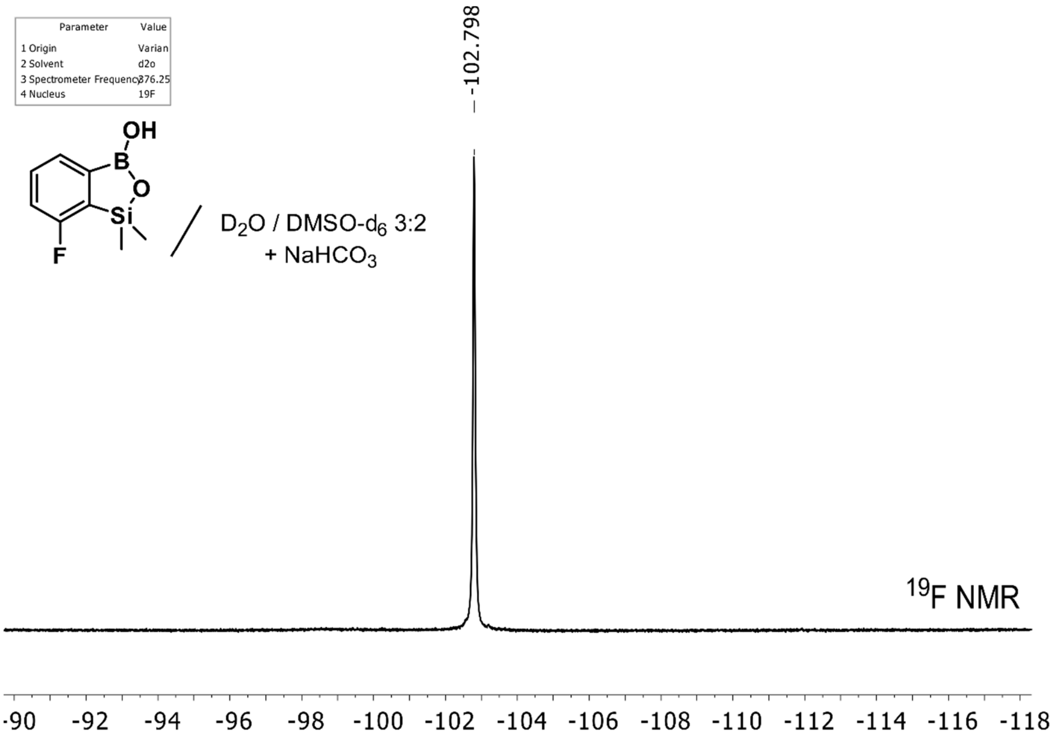

Figure 4. ^{19}F NMR spectrum (376 MHz, D_2O + DMSO-*d*$_6$ 3:2) of compound **1** in the presence of $NaHCO_3$.

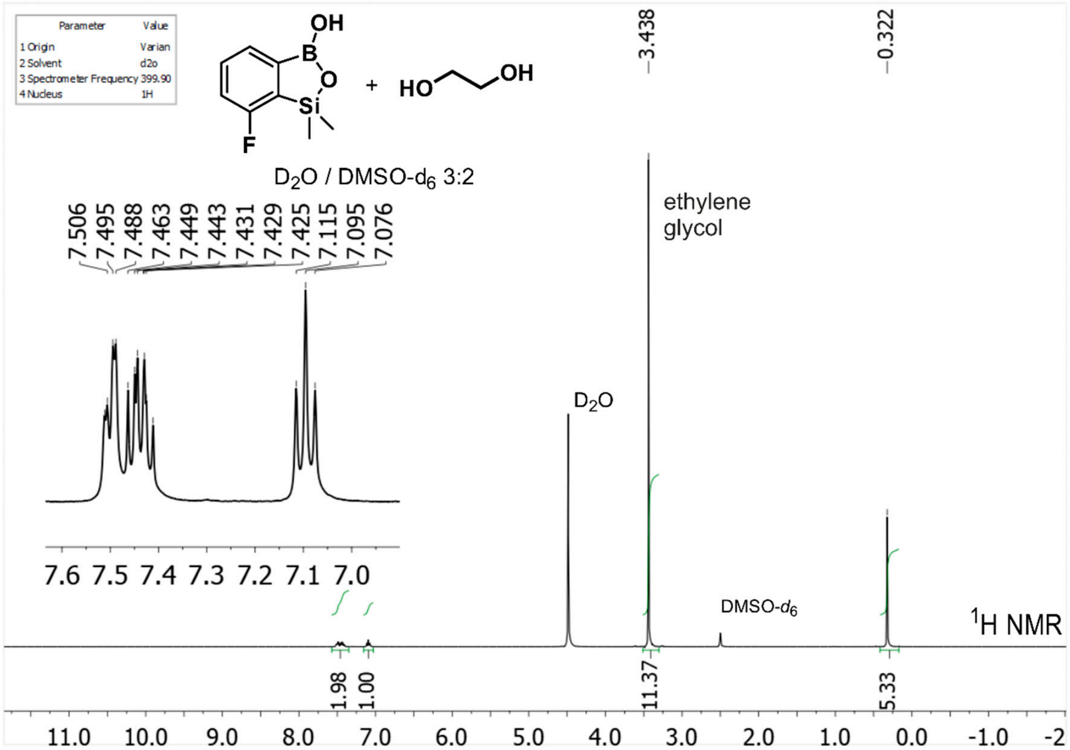

Figure S5. ^{1}H NMR spectrum (400 MHz, D_2O + DMSO-*d*$_6$ 3:2) of **1** in the presence of ethylene glycol.

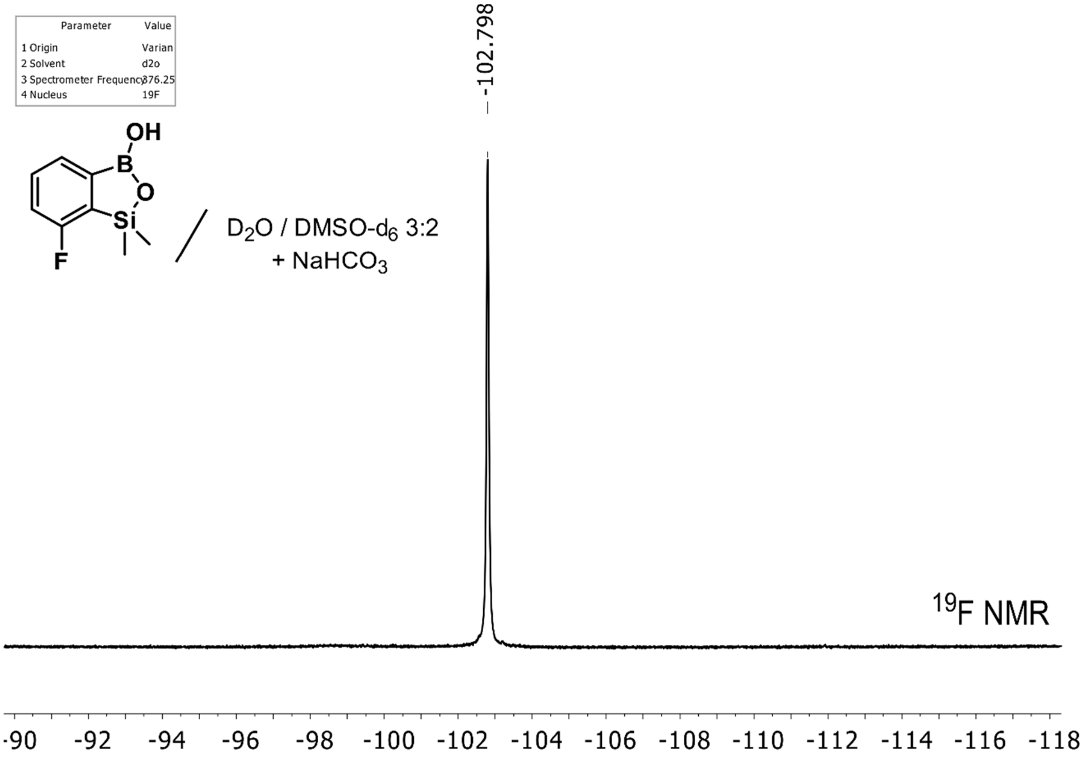

Figure S6. ^{19}F NMR spectrum (376 MHz, D_2O + DMSO-d_6 3:2) of **1** in the presence of ethylene glycol.

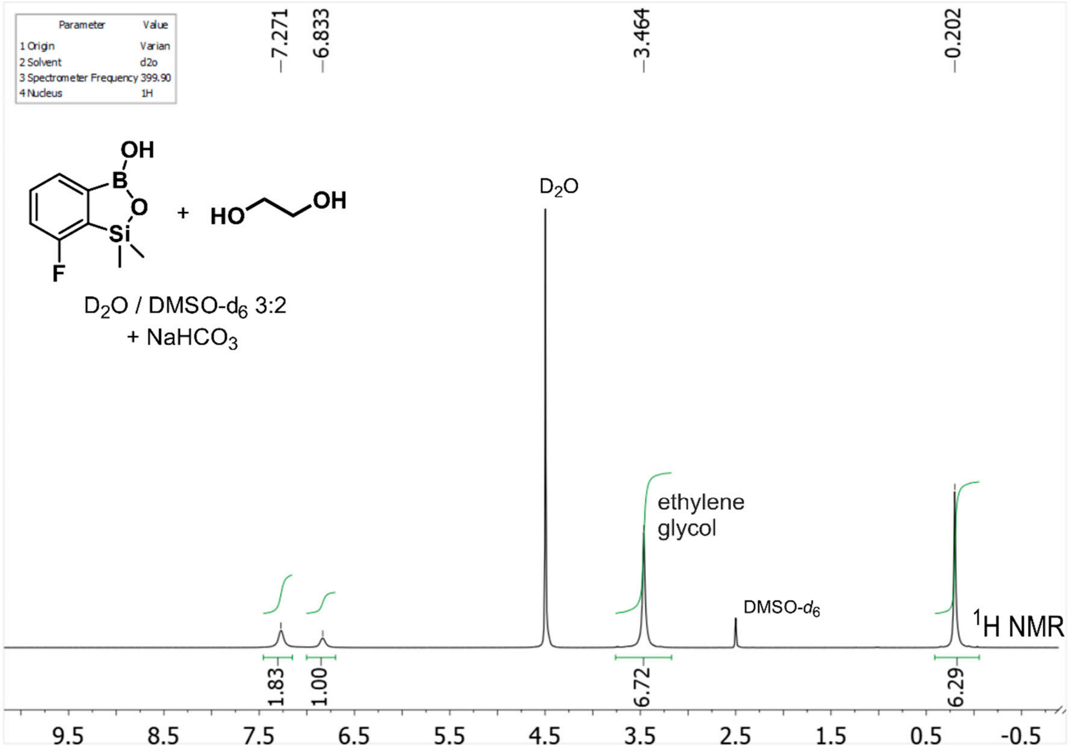

Figure S7. ^{1}H NMR spectrum (400 MHz, D_2O + DMSO-d_6 3:2) of **1** in the presence of ethylene glycol and $NaHCO_3$.

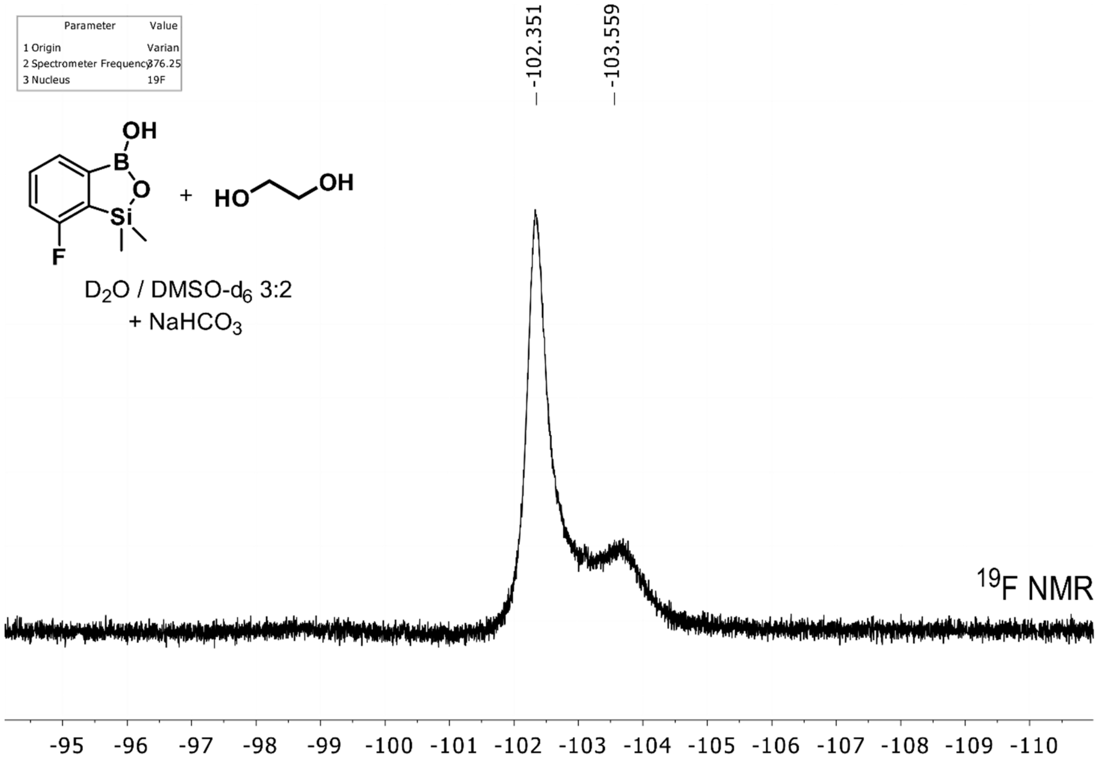

Figure S8. ^{19}F NMR spectrum (376 MHz, D_2O + DMSO-*d*$_6$ 3:2) of **1** in the presence of ethylene glycol and $NaHCO_3$.

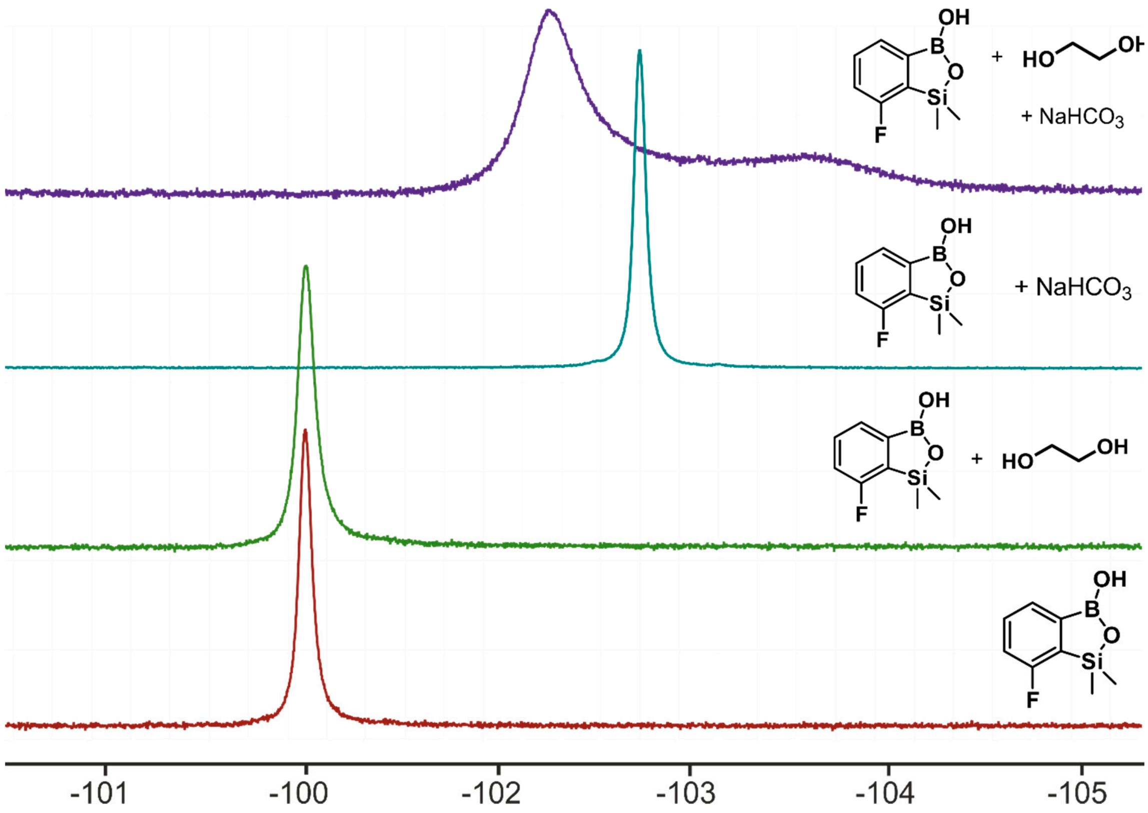

Figure S9. Overlay of ^{19}F NMR spectra (376 MHz, D_2O + DMSO-d_6 3:2) of **1** in different conditions.
